# Periodontitis as a risk for oral cancer: a case–control study

**DOI:** 10.1186/s12903-021-01998-y

**Published:** 2021-12-15

**Authors:** György Komlós, Katalin Csurgay, Ferenc Horváth, Liza Pelyhe, Zsolt Németh

**Affiliations:** 1 Department of Oro-Maxillofacial Surgery and Stomatology, Faculty of Dentistry, Semmelweis University, Mária str 52, 1085 Budapest, Hungary; 2 Department of Public Health, Faculty of Medicine, Semmelweis University, Nagyvárad Sq. 4, 1089 Budapest, Hungary; 3 Independent Researcher, Budapest, Hungary

**Keywords:** Periodontal disease, Oral cancer, Risk factors

## Abstract

**Background:**

The aetiology of oral cancer is multifactorial, as various risk factors (genetics, socioeconomic and lifestyle factors) contribute to its development. Data in the literature suggest that people with periodontal disease have an increased risk of developing oral cancer, and the severity of periodontitis correlates with the appearance of oral squamous cell carcinoma. The aim of this study was to revise the non-genetic risk factors that may influence the development of OC, while focusing on the dental and periodontal status and OH.

**Methods:**

Two hundred patients (hundred diagnosed with oral cancer and hundred without oral cancer) were enrolled in our case–control study, to evaluate the association between oral cancer and the presence and severity of periodontitis, while examining several risk factors that might be responsible for oral cancer formation. A questionnaire customised for oral cancer patients was used to obtain the socioeconomic and lifestyle risk factors that may influence the development of oral squamous cell carcinoma. The dental and periodontal status along with the level of oral hygiene was recorded quantitatively. The chi-square and Mann–Whitney tests and logistic regression were used for the statistical analysis.

**Results:**

By considering both the case and the control groups, a significant correlation was found between the incidence of oral cancer and some socioeconomic factors and lifestyle habits, such as the sex, age, education and alcohol consumption of an individual. The mean value of the Silness-Löe plaque index was significantly higher in the case population. The number of completely edentulous patients was higher among the oral cancer population. The incidence of oral cancer was 57.1% in patients with periodontal disease. In comparison, the incidence of oral squamous cell carcinoma was only 28.6% among the patients without periodontitis. Most of the oral cancer patients (72.1%) had stage 4 periodontitis. On the other hand, the vast majority of the control group (51.6%) had stage 2 periodontitis.

**Conclusion:**

Periodontitis can be an individual risk factor for oral cancer development. Periodontally compromised individuals should be strictly monitored, especially those with *severe periodontitis* and coexisting lifestyle risk factors. Maintaining their periodontal health in at-risk patients can minimize cancer risks.

## Background

Cancers of the head and neck region (HNC) accounts for more than 650,000 cases and 330,000 deaths annually worldwide [1]. One subtype of HNC involving the lips and the oral cavity is OC, and its most common histology is squamous cell carcinoma (SCC). According to the 2018 database of *Globocan*, cancer of the lip and the oral cavity is the 16th most common form, with approximately 354,000 new cases per year worldwide [2]. The *Hungarian Cancer Registry* reported 1449 patients treated with OC in *Hungary* in 2017 [3]. It has already been suggested that the aetiology of OC is multifactorial. Alcohol consumption and smoking are two of the most important risk factors [4]. In most epidemiological studies, heavy drinking is associated with a significantly elevated risk that is independent of smoking [5]. A case–control study published in 2018 revealed that there is an inverse association between the risk of oral cavity SCC and low education and income satisfaction [6]. Associations were observed between the oral hygiene (OH) indicators and the appearance of OC, independent of alcohol consumption and cigarette smoking [7]. The evidence in the literature clearly proves that OC is more common in males than in females [8]. Recent data showed that periodontitis associated with bad OH can be a causative factor of OC [9]. There is a link between the incidence of oral SCC and ageing, which is a common risk factor for periodontal disease (PD) [10],^11^. Some large prospective trials showed that people with periodontal disease had an increased risk of developing OC [12]^13^. The currently available data in the literature strongly support that periodontal disease can be an independent risk factor for OC [14]. In a cohort study, *Tezal *et al*.* found an increased incidence of oral tumours in patients with greater than 1.5 mm clinical attachment loss (CAL) [15]. Based on the observation of Al-Hebshi et al., periodontal pathogens such as *Fusobacterium nucleatum* and *Porphyromonas gingivalis* are often isolated from oral neoplasms [16]*.* According to a huge bibliographic research carried out selecting articles published until 2020, *Porphyromonas gingivalis, a member of red complex consortium of Gram-negative anaerobs* was suggest to play an important role in OC development [17]. The currently available evidence also indicates that periodontal lesions contain important inflammatory mediators that may be associated with carcinogenesis [18, 19]. In periodontal disease a constant systemic inflammation is sustained, increasing plasma levels of acute phase proteins and proinflammatory cytokines [20]. The development of a malignant lesion can be associated with inflammation itself, peculiarly as it causes oxidative damage on the cell’s DNA [21]. The link between OC and tooth loss, or periodontal disease, was evaluated in some studies. Most of them found a significantly increased risk of OC associated with increased tooth loss or other parameters of periodontitis even after the parameters were adjusted for the use of tobacco and alcohol [22]. Recent evidence also suggests that the alteration of the oral microbiome in periodontal diseases may create an environment, that subserves the development of oral SCC. Therefore, the severity of periodontitis may be associated with the evolution of oral carcinoma [23]. The aim of this study was to revise the non-genetic risk factors that may influence the development of OC, while focusing on the dental and periodontal status and OH.

## Methods

In this case–control study, a group of patients with OC was compared to a group of patients who did not have OC. The presence and the *severity* of periodontitis in correlation with the appearance of OC was examined. Patients who were 18–90 years old and were examined and/or treated at the Department of Oro-Maxillofacial Surgery and Stomatology at the Semmelweis University were recruited and enrolled in this research. In this study, 200 patients were included altogether. Patients were selected independently of their periodontal status. The case group consisted of 100 patients who had a histologically confirmed diagnosis of OSCC. In contrast, the control group consisted of the other 100 patients who did not have OC in their medical history. Patients were selected regardless of the presence of bad habits (smoking and alcohol consumption). In the case group, patients were selected with a histologically confirmed diagnosis of oral SCC (in the upper lip, lower lip, bucca, gingiva, tongue, sublingual region or palate) independent of their sex, age and the localization/extent of the malignant lesion. All the individuals with potentially malignant disorders or cancers other than SCC were excluded from this population. In addition, the exclusion criterion consisted of all cancers whose appearance differed from the previously named sites of the head and neck region. Patients were not excluded with SCC that appeared on multiple sites of the oral cavity at the same time. Patients enrolled in the control group were seen at our department for the treatment of other, non-malignant diseases. They were randomly selected to be added to this population, independent of their age and sex. A computer-generated randomization scheme was used to allocate patients from an existing database of the Department of Oro-Maxillofacial Surgery and Stomatology at the Semmelweis University to the case and control groups in between January of 2017 and June of 2019. Out of patients appearing within a given half a year we randomly selected 20 with OC and 20 without OC. This is how we could ensure 100–100 patients in the two groups within the time frame of 2.5 years. Patients who had OC in their medical history were excluded from the control group. Patients who were illiterate and patients who were unable to answer the questions of the survey by themselves were not selected to participate in this study. All the individuals who did not fill out the questionnaire successfully were excluded from the study. Multivariable examination was performed with a questionnaire customised for OC patients. The questionnaire was designed in cooperation with the Department of Public Health at Semmelweis University. The classification of certain groups according to the observed risk factors is shown in Table 2. In the course of clinical examination, the dental status of the patients was recorded with the DMF-index in compliance with the criteria of the *World Health Organisation* (WHO) [24]. The stage of OH was defined by using the Silness-Löe plaque index (SLPI). For the evaluation of the periodontal state, the probing pocket depth (PPD) and clinical attachment loss (CAL) were calculated from the measurements performed on 6 points next to each tooth (the buccal, mesiobuccal, distobuccal, lingual/palatal, distolingual/distopalatal, and mesiolingual/mesiopalatal points). The average of the observed bleeding on probing (BOP) data was calculated. A *Williams* periodontal probe was used for the measurements. To eliminate measurement errors, all assessments were performed by the same dentist, who was aware of the study protocol. The severity of periodontitis was determined according to the classification created by the *World Workshop on the Classification of Periodontal and Peri-Implant Diseases and Conditions* in 2017 [25]. The criteria of determining the severity of periodontitis are shown in Table 1.

**Table 1:** Staging of periodontitis

	Periodontitis	Stage I	Stage II	Stage III	Stage IV
Severity	Interdental CAL (at the site of greatest loss)	1–2 mm	3–4 mm	≥ 5 mm	≥ 5 mm
	Tooth loss (due to periodontitis)	No tooth loss		≤ 4 teeth	≥ 5 teeth

Criteria to determine the severity of periodontitis according to the *World Workshop on the Classification of Periodontal and Peri-Implant Diseases and Conditions* in 2017

The exact OC localisation was registered. To ensure the anonymity of our study participants, the patients individually attached the registration form containing the measured and calculated data of the oral status of the patient to the completed questionnaires and placed them into a labelled and sealed container. The container was opened when the study population reached 200 individuals. Data were registered by GYK. Patients were informed about the aim and the course of the study orally and in written form. Written informed consent was signed by all the participants. The essential oncologic treatment of the patients was taken place at the Department of Oro-Maxillofacial Surgery and Stomatology at the Semmelweis University. For conservative dental treatment and the treatment of the periodontal disease, patients were referred to their dentists / a specialist. The necessary ethical approval for our study was approved by the *Semmelweis University Regional and Institutional Committee of Science and Research Ethics* (reference number: 252/2016; date of registration: 16.12.2016). Using *SPSS Statistics 22* (IBM Corporation, Armonk, New York, USA) programme package and *Microsoft Office Excel 2007* (Microsoft Corporation, Redmond, Washington, USA), *chi-square tests, Mann–Whitney tests and logistic regression* were performed for the statistical analysis. The chi-square test is a statistical test that can be used to analyse the relationship between two quality variables. It was used in case of nominal and ordinal variables. In case of two scale type variables, *Mann–Whitney tests was used to detect significant differences between them.* A *p* value less than 0.05 was accepted as statistically significant.

## Results

### Evaluation of the socioeconomic and lifestyle risk factors

The vast majority of oral SCCs were detected on the tongue (27%). This was followed by the cancer of the sublingual region and the gingiva (18% and 11%). Lower OC values were distributed between the lips, palate, and cheeks. Six cases were registered in which cancer of the sublingual region and the tongue appeared together at the same time (6%). The study population was predominantly married male individuals over the age of 50.

Most of the individuals had finished secondary school (62%), and they were currently working (53%). Patients of the case group were mostly above the age of 50. The division of male and female patients in the case population was 66:33. One patient did not keep record his or her sex. Sixty-three percent of the patients in the control group were under the age of 50, and the division between sexes was 48% males and 52% females. A significant correlation was found between the development of OC and the sex of the patient (*p* = 0.008), and between the development of OC and the age of the patient (*p* = 0). There were significant differences in the age (*p* = 0) and sex (*p* = 0.01) between the case and control groups. There was no significant correlation detected in between age and sex (*p* = 0.055). A correlation was recorded between the marital status and the appearance of the oral SCC. The vast majority of the case group patients were married (49%), in contrast to the control group, where most of the individuals were single (45%). A correlation was found in between the development of OC and the educational level. The proportion of less-educated patients was similar in the two groups; however, the incidence of the oral SCC was almost twice as high in the less-educated individuals (67.6%/32.4%); there was an inverse relationship for highly educated patients. In the case group, the development of OC was related to a lower education. Patients of the entire population described their income as sufficient (61.5%). There was no considerable difference in the level of income among the two groups. In the case group, there was no correlation between the smoking habits and the incidence of OC. A total of 44.4% of the patients in the case group were considered current smokers. Among the case group individuals that were current smokers, the patients who smoked less than 20 cigarettes a day had a higher rate of OC. There was no significant correlation between passive smoking and the development of OC. On the other hand, there was a significant correlation between the development of oral SCC and the amount of alcohol intake (*p* = 0.026). Heavy drinking was an independent risk factor for the development of OC in our study. The examined socioeconomic risk factors and lifestyle habits are shown in Table 2.

**Table 2:** The examined socioeconomic risk factors and lifestyle habits in the case and the control groups are shown. *p* Value was calculated by *chi-square tests.* The ‘bold’ *p* values show significant differences

		Case	Control	*p* value
Age (%)	Above 50	87	37	**0.00**
	Below 50	13	63	
Sex (%)	Male	66	48	**0.01**
	Female	33	52	
Marital status (%)	Single	14	45	**0.00**
	Married	49	39	
	Widow(er)	18	4	
	Divorced	19	12	
Occupation (%)	Currently working	43	63	**0.00**
	Unemployed	4	4	
	Housewife/homemaker	2	1	
	Retired	49	20	
	Disabled	2	5	
	Student	0	7	
Education (%)	Elementary school	23	11	**0.03**
	Secondary school	62	63	
	High school	15	26	
Income (%)	Good	22	24	0.15
	Proper	58	66	
	Low	20	10	
Smoking habit (%)	Current smoker	47	43	0.16
	Has not smoked for Less than a year	13	8	
	Has not smoked for More than a year	22	18	
	Never	18	31	
Passive smoking (%)	At home	66	63	0.73
	Outside of home	60	58	0.88
Alcohol consumption (%)	Daily	18	6	**0.03**
	Weekly	24	19	
	Monthly	13	24	
	Never	45	51	

### Evaluation of the oral status

**Fig. 1:**
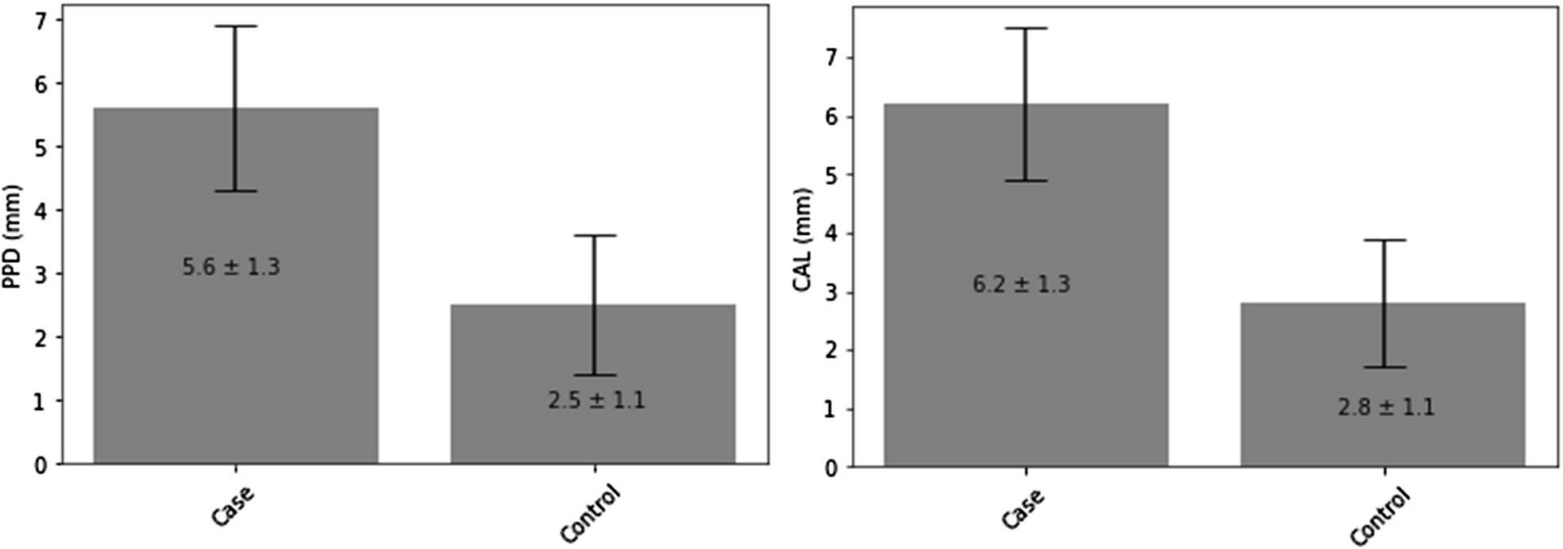
The division of PPD and CAL value in the study population

Analysis of the dental status showed there were more completely edentulous patients in the case group than in the control group. A total of 88.2% of the completely edentulous patients had OC. The rate of filled teeth (F) was significantly higher in the control group than in the case group, in which the rate of missing teeth (M) was higher. Regarding the number of decayed teeth (D), no significant difference was detected between the two groups. The mean value of DMFT in the case group was 21.65 ± 8.46, in contrast to the case group, in which it was 14.18 ± 8.26. A significant correlation was detected between the incidence of OC and periodontitis. The appearance of oral SCC was 57.1% among patients with periodontitis. On the other hand, the incidence of OC was only 28.6% among patients without periodontal disease. A significant correlation was observed between the severity of PD, the periodontal stage, and OC. Most of the OC patients had stage 4 periodontitis (72.1% in the case group), in contrast to the control group, in which most of the individuals had stage 2 periodontitis (51.6%). In comparing the case and the control groups, the incidence of OC increased with the severity of periodontitis: the appearance of OC was more common in patients with higher levels of periodontal disease. There was a significant difference in the clinical attachment loss (CAL) and probing pocket depth (PPD) between the two groups examined. Both of the previously named values were higher in the case group. The mean value of CAL in the case group was 6.2 ± 1.3. This value was 2.8 ± 1.1 in the control group. The mean value of PPD among patients with oral SCC was 5.6 ± 1.3, and it was 2.5 ± 1.1 in the non-cancer population (Fig. 1.)

There was no significant difference between the two groups in the BOP data (*p* = 0.09). The mean BOP value among the OC population was 44.9 ± 30.4, and it was 31.4 ± 23.2 in the control population. The index describing oral hygiene (Silness-Löe plaque index) showed a significant difference in between the case and the control groups. The mean value of SLPI was 2.6 ± 0.8 in the case group, and in contrast, it was 1.6 ± 0.9 in the control group. The oral status in the case and the control group is compared in Table 3. Logistic regression analysis proved that the rate of periodontitis and its severity remained statistically significant even after adjusting for smoking and alcohol consumption. According to our study, periodontitis appears to be an individual risk factor for OC development. The influence of different smoking habits and alcohol consumption on the oral health status is shown in Table 4. The highest values were detected among the patients with daily alcohol consumption who did not smoke for more than a year.

**Table 3:** Comparison of the oral status in the case and in the control group

		Case	Control	*p* value
Periodontal stage (%)	I	0	8	**0.00**
	II	0	17	
	III	14	4	
	IV	47	2	
CAL (mm)		6.2 ± 1.3	2.8 ± 1.1	**0.00**
PPD (mm)		5.6 ± 1.3	2.5 ± 1.1	**0.00**
BOP (%)		44.9 ± 30.4	31.4 ± 23.2	0.09
SLPI		2.6 ± 0.8	1.6 ± 0.9	**0.00**
DMFT index		21.65 ± 8.46	14.18 ± 8.26	**0.00**

The oral status of the patients in the case and the control group is compared according to periodontal stage, clinical attachment loss (CAL), periodontal pocket depth (PPD), bleeding on probing (BOP), Silness-Löe plaque index and DMFT index (decayed, missing and filled teeth). *p* value was calculated by *chi-square tests* in case of periodontal stage (%), and it was calculated by *Mann–Whitney tests* in all the other cases. The ‘bold’ *p* values show significant differences

**Table 4:** The oral health status of the 200 patients with information on smoking and alcohol consumption

			Smoking habit			
			Current smoker	Has not smoked for less than a year	Has not smoked for more than a year	Never
Alcohol consumption	Daily	Stage	3.0 ± 0.9	n.d	**4.0 ± 0.0**	**4.0 ± n.d**
		BOP	45.4 ± 24.4	n.d	**75.7 ± 22.7**	25.0 ± 35.4
		SLPI	**2.3 ± 0.9**	n.d	**3.0 ± 0.0**	1.3 ± 1.1
		DMFT	21.9 ± 9.9	**28.0 ± n.d**	24.6 ± 5.0	8.5 ± 10.6
	Weekly	Stage	2.9 ± 1.1	2.5 ± 2.1	**3.3 ± 0.8**	2.3 ± 1.5
		BOP	37.3 ± 27.9	28.4 ± 16.6	**38.0 ± 19.1**	27.6 ± 26.0
		SLPI	**2.3 ± 0.8**	2.1 ± 0.6	2.0 ± 0.8	1.1 ± 1.0
		DMFT	20.4 ± 10.4	**20.9 ± 7.0**	19.2 ± 9.9	12.4 ± 9.1
	Monthly	Stage	2.5 ± 1.2	**4.0 ± n.d**	3.0 ± n.d	n.d
		BOP	**36.7 ± 14.5**	29.6 ± 32.2	6.1 ± 5.5	28.5 ± 23.8
		SLPI	1.9 ± 0.9	**2.1 ± 1.1**	1.4 ± 0.8	1.2 ± 1.0
		DMFT	14.9 ± 7.6	**18.6 ± 9.9**	15.2 ± 6.2	10.8 ± 8.2
	Never	Stage	3.0 ± 1.1	**4.0 ± n.d**	2.0 ± 1.2	3.4 ± 0.9
		BOP	32.5 ± 28.9	38.8 ± 30.2	31.4 ± 26.0	**42.0 ± 29.4**
		SLPI	1.8 ± 0.8	1.8 ± 1.1	1.7 ± 1.1	**2.0 ± 0.9**
		DMFT	17.4 ± 7.5	18.1 ± 8.3	**20.4 ± 9.5**	17.6 ± 10.2

^*^n.d.: no data

The oral health status of the 200 patients is visible in this table with information on smoking and alcohol consumption. In all row, the maximum values were bold. The worst oral health status was detected among the patients with daily alcohol consumption and current smoking habit

## Discussion

Ever since *Seymour* stated in 2010 that poor oral hygiene may affect the risk of OC, a link between PD and the development of OC has been suspected [26]. Periodontitis is an inflammatory process affecting the supporting structure of the teeth, including the gingiva, periodontal ligaments and the bone [27]. Elevated plasma levels of several proinflammatory cytokines, acute phase proteins and proteinases can be observed in periodontal disease [28]. The development of a malignant lesion can be associated with inflammation itself, peculiarly as it causes oxidative damage on the cell’s DNA [21]. The recent studies of *Gopinath *et al. *and Geng *et al. on the development of OC suggests that there is a direct link between periodontal pathogens and the carcinogenesis [29, 30]. In the comprehensive systematic review and meta-analysis performed by *Gopinath *et al. in 2020, it was stated, that periodontitis appears to be an individual risk factor for HNC development [14]. Our analysis showed a significant correlation between the incidence of OC and periodontitis, which supports the systematic review published by Fawad et al. in 2016 [11]. As stated by Tezal et al. in 2005, the diversity of study designs, the variable study population and the different measuring procedures represent limitations of the studies published on periodontal disease as a risk factor for OC [15]. The publications on this topic use non-quantitative methods for establishing the diagnosis of PD. The existence of periodontitis was defined by the partial periodontal indexes (CPITN for example), tooth loss, and self-reported periodontal evaluation [31]. Our study involves quantitative measurements and clearly supports the observation of *Qian *et al., that there is an association between tooth loss and the consequential bone resorption and the risk of oral SCC [32]. A high number of completely edentulous patients was registered in the case group. In our whole study population, 88.2% of the completely edentulous patients had OC. During our statistical analysis, we did not connect the edentulousness to the periodontitis, as there was no information existing about the reasons for tooth loss. CAL and PPD were used to evaluate the existence/lack of periodontitis and its severity (periodontal stage), ensuring a more reliable result. There is a clear positive link between the severity of periodontitis and the incidence of OC. In the control group, all the PPD and CAL values were higher than those in the case group. This supports the hypothesis of Sahingur et al. that the severity of PD and changes in the oral microbiome creates a suitable environment for the development of oral SCC [23]. A study by Hormia et al. also suggests that periodontal pockets may be considered reserves for the cytomegalovirus, HPV and Epstein-Barr virus, which are agents that may be associated with OC [19]. Moreover, according to Colotta et al., periodontal lesions include some inflammatory mediators (for example, IL1-ß and TNF-α) associated with carcinogenesis [18]. Alcohol consumption, ageing, smoking and poor oral hygiene are important risk factors for periodontitis [33, 34, 35]. It should be noted that the same factors can also be associated with carcinogenesis in the oral cavity [5]. It has already been proven by Jeman et al. that the incidence of OC is higher in developing countries. In these countries, the infrastructure of the health care system, and thus preventive care, is underdeveloped [36]. According to Babiker et al., more structured preventive programmes would reduce the rate of morbidity and mortality associated with OC [37]. In our study, an association was observed between the incidence of oral SCC and the low education level. Poor levels of OH and smoking can be related to the low education levels [38]. Analysing the effect of smoking habits and alcohol consumption on the patients’ oral health, it was observed that alcohol consumption has a bigger impact on the oral health status. Our findings correlate to published data [39, 40]. According to the observation of Hashim et al., good oral hygiene may reduce the risk of OC [7]. The degree of OH represented by Silnes-Löe plaque index was significantly higher in the case population than in the control population. Our study supports the idea that regular dental visits and sufficient OH can reduce the incidence of OC [9]. In our study there was a significant difference in age (*p* = 0) and sex (*p* = 0.02) between the control and case populations. In this manner, both of these factors influence the development of OC. There was no statistical correlation between the age and sex (*p* = 0.055) of the patients, although a higher case number may have changed these marginal values. In our study, there was a link between marital status and the incidence of OC. Forty-nine percent of the case group was married in contrast to the control group, in which 45% of the individuals described themselves as single. In most epidemiological studies, heavy drinking is associated with a significantly increased risk of oral SCC independent of from cigarette smoking. According to the observation of Goldstein et al. the risk increases with increased alcohol intake [41]. There was a significant correlation in between the development of oral SCC and the alcohol intake level (*p* = 0.026), indicating that heavy drinking was an independent risk factor for the development of OC in our study. In addition, some oral microorganisms metabolize alcohol to carcinogenic acetaldehyde, which may explain the association between alcohol intake, poor oral hygiene and carcinogenesis [42]. Based on the data from the currently available literature, a directly proportional effect occurs between the pack years of tobacco smoking and the risk of OSSC [43]. Contrary to the expected result, there was no significant correlation between the smoking habits and the incidence of oral SCC in our study. Forty-four percent of the examined patients in our study were current smokers. Among the current smoker of OC cancer patients, the majority of individuals smoked less than one pack (20 pieces) per day. PD and OC have many common risk factors; however, periodontitis emerges as an individual risk factor in the background of the oral SCC development [44, 45]. In our study, it was proven with logistic regression analysis that the rate of periodontitis and its severity remained statistically significant even after adjusting for smoking and alcohol consumption. The precise, quantitative measurements for evaluating the periodontal status can be considered as the strength of our study. According to the available articles of the literature, the limitation of the studies published on the same topic was defined to be the inaccurate measurement procedures. The small number of cases is a limitation of our study and a larger study population may alter the results obtained.

## Conclusion

Poor oral hygiene and the ensuing accumulation of plaque accumulation results in a chronic inflammatory process, creating an environment that promotes the development of OC. Our study supports the hypothesis that periodontitis is an individual risk factor for OC. The risk of the OSCC increases at more severe stages of periodontitis. Periodontal disease is a chronic inflammatory condition that may be prevented with regular dental visits and with the maintenance of the OH. Dentists play an important role in preventing oral cancer by evaluating the socioeconomic status of each patient and appraising their lifestyle and habits. Motivating patients at risk of OC to maintain sufficient OH is crucial and easily achievable. Preserving the periodontal health and monitoring the individuals with lifestyle risk factors who are periodontally compromised may minimize the risk of OC.

## Data Availability

The datasets used and/or analysed during the current study are available from the corresponding author (György Komlós, komlos.gyorgy@dent.semmelweis-univ.hu) on reasonable request.

## Abbreviations

BOP: Bleeding on probing
CAL: Clinical attachment loss
CPITN: Community Periodontal Index for Treatment Needs
DMFT: Decayed, missing and filled teeth
HNC: Head and neck cancer
OC: Oral cancer
OH: Oral hygiene
OSCC: Oral squamous cell carcinoma
PD: Periodontal disease
PPD: Periodontal pocket depth
SCC: Squamous cell carcinoma
SLPI: Silness-Löe plaque index
WHO: World Health Organisation
